# Monitoring Risk Factors and Improving Adherence to Therapy in Patients With Chronic Kidney Disease (Smit-CKD Project): Pilot Observational Study

**DOI:** 10.2196/36766

**Published:** 2022-11-15

**Authors:** Antonio Vilasi, Vincenzo Antonio Panuccio, Salvatore Morante, Antonino Villa, Maria Carmela Versace, Sabrina Mezzatesta, Sergio Mercuri, Rosalinda Inguanta, Giuseppe Aiello, Demetrio Cutrupi, Rossella Puglisi, Salvatore Capria, Maurizio Li Vigni, Giovanni Tripepi, Claudia Torino

**Affiliations:** 1 Institute of Clinical Physiology National Research Council Reggio Calabria Italy; 2 Nephrology Unit Grande Ospedale Metropolitano Bianchi Melacrino Morelli Reggio Calabria Italy; 3 Immedia Società per Azioni Reggio Calabria Italy; 4 Mercuri Informatica Reggio Calabria Italy; 5 Department of Engineering University of Palermo Palermo Italy

**Keywords:** SMIT-CKD, mHealth, eHealth, CKD, therapy adherence, risk factor, kidney, adherence, integrated system, health app, monitoring, cardiology, cardiac, renal, chronic kidney disease, cardiovascular, mobile health, mobile app

## Abstract

**Background:**

Chronic kidney disease is a major public health issue, with about 13% of the general adult population and 30% of the elderly affected. Patients in the last stage of this disease have an almost uniquely high risk of death and cardiovascular events, with reduced adherence to therapy representing an additional risk factor for cardiovascular morbidity and mortality. Considering the increased penetration of mobile phones, a mobile app could educate patients to autonomously monitor cardiorenal risk factors.

**Objective:**

With this background in mind, we developed an integrated system of a server and app with the aim of improving self-monitoring of cardiovascular and renal risk factors and adherence to therapy.

**Methods:**

The software infrastructure for both the Smit-CKD server and Smit-CKD app was developed using standard web-oriented development methodologies preferring open source tools when available. To make the Smit-CKD app suitable for Android and iOS, platforms that allow the development of a multiplatform app starting from a single source code were used. The integrated system was field tested with the help of 22 participants. User satisfaction and adherence to therapy were measured by questionnaires specifically designed for this study; regular use of the app was measured using the daily reports available on the platform.

**Results:**

The Smit-CKD app allows the monitoring of cardiorenal risk factors, such as blood pressure, weight, and blood glucose. Collected data are transmitted in real time to the referring general practitioner. In addition, special reminders improve adherence to the medication regimen. Via the Smit-CKD server, general practitioners can monitor the clinical status of their patients and their adherence to therapy. During the test phase, 73% (16/22) of subjects entered all the required data regularly and sent feedback on drug intake. After 6 months of use, the percentage of regular intake of medications rose from 64% (14/22) to 82% (18/22). Analysis of the evaluation questionnaires showed that both the app and server components were well accepted by the users.

**Conclusions:**

Our study demonstrated that a simple mobile app, created to self-monitor modifiable cardiorenal risk factors and adherence to therapy, is well tolerated by patients affected by chronic kidney disease. Further studies are required to clarify if the use of this integrated system will have long-term effects on therapy adherence and if self-monitoring of risk factors will improve clinical outcomes in this population.

## Introduction

Chronic kidney disease (CKD) is a recognized major public health problem, with about 13% of the general adult population falling into one of the 5 stages identified by the Kidney Disease Outcome Quality Initiative classification [1]. Its prevalence increases to 15% to 30% in older persons, and exceeds 50% in patients with cardiovascular and metabolic comorbidities [1,2].

Additionally, these patients have an almost uniquely high risk of death and cardiovascular disease, with a rate of cardiovascular events strongly associated with the level of renal function [3]. The pathogenic mechanisms underlying the close relationship between kidneys and the cardiovascular system are not fully elucidated; however, the direct involvement of diabetes mellitus, arterial hypertension, and excess body weight in the high frequency of cardiovascular events both in renal failure and ischemic heart disease has been clarified [4-6]. More recently, reduced adherence to therapy has been recognized as an additional risk factor for cardiovascular morbidity and mortality in renal patients [7,8]. It is estimated that patients affected by CKD, especially those in the late stages, take on average 10 different drugs [9]. In these patients, low adherence may be due to the difficulty in reminding days and time of medicine intake, rather than the unwillingness to take medications [10-13], and this may explain the number of apps for medication monitoring in the major app stores [14,15].

The use of mobile apps for self-management in long-term conditions is not novel [16], and their number is increasing exponentially with the increasing use of mobile phones. A review published by Timmers et al [17] in 2020 showed that the use of smartphones for patient education improves medication or treatment adherence and clinical outcomes. Several mobile apps for the management of chronic conditions, such as diabetes and high blood pressure, are available in Google Play and the App Store [18-20]. Some of them are designed to monitor and correct patient behavior in order to reduce modifiable cardiovascular risk factors [21]; other solutions implement home-based rehabilitation programs for critically ill cardiovascular patients [22]. However, the large majority of these apps do not have medication monitoring as the main purpose [23], are not specifically focused on CKD patients [14,24,25], or are dedicated to patients in the late stages (eg, dialysis patients) [26].

With this in mind, the Smit-CKD project aimed at developing an integrated system designed for general practitioners (GPs) and patients consisting of a web-based platform (Smit-CKD server) and an app (Smit-CKD app), with the aim of improving medication regimen compliance and educating patients in self-monitoring of the most common risk factors for CKD and cardiovascular disease.

## Methods

### Design of the Smit-CKD Server and Smit-CKD App

As previously described, the growing coverage of the mobile cellular network and increased interest in mobile health (mHealth; the use of mobile and wireless technologies to support the achievement of health objectives) led us to conclude that an app specifically designed for the CKD population could be a good solution to improve adherence to therapy and decrease modifiable cardiorenal risk factors. In order to define the general architecture of the system, an in-depth bibliographic search was conducted with the aim of identifying the main risk factors for the progression of renal disease and clinical outcomes. Renal function is closely related to cardiovascular risk [3], so we started with the Framingham Heart Study [27], deriving a minimum set of predictors of mortality and cardiovascular events in the general population (age, sex, smoking, diabetes, previous cardiovascular events, cholesterol, high blood pressure) [28-30]. Other risk factors, such as BMI, an indicator of overweight and obesity [31,32], education level [33], and marital status [34] were subsequently added because of their association with mortality in the general population. Combining bibliographic research with good clinical practice, we defined the set of variables to be collected in the platform during the baseline and follow-up visits. This set includes personal data, anamnesis, education level, marital status, work activity, laboratory tests, somatometric data, blood pressure, and medications.

The next step consisted in the choice, among all the variables included the platform, of a minimum set of easily monitorable factors to be included in the app (ie, blood pressure, weight, diabetes [if any], and adherence to therapy) [35].

Regarding the frequency of blood pressure, blood glucose, and weight measurements, current guidelines for the management of blood pressure and diabetes in patients with chronic kidney disease were used.

### Development of the Smit-CKD Server and Smit-CKD App

The software infrastructure for both the Smit-CKD server and Smit-CKD app was developed using standard web-oriented development methodologies, choosing open source tools when available, and MySQL as a database server. To ensure compatibility with the two major app stores, the choice was oriented toward platforms allowing the development of a multiplatform mobile app starting from a single source code.

To make the app suitable for the target audience (ie, patients with chronic kidney disease, in most cases elderly), we chose a user friendly interface, limiting the amount of information collected and user/interface interactions.

### Design and Validation of Questionnaire to Measure Adherence to Therapy

The questionnaire to measure adherence to therapy used in the SMIT-CKD project was created after a literature search using *therapy* AND *adherence* AND *questionnaire* as keywords. All questionnaires resulting from this research were analyzed in order to create a new questionnaire that suited the needs of this study. Specifically, we examined the 4- and 8-question versions of the Morisky Medication Adherence Scale [36] and the Renal Treatment Satisfaction Questionnaire [37], the latest specially designed for CKD patients. Furthermore, we included questions on marital status, education level, and income since they are known risk factors for the progression of various chronic diseases [33,34,38] and may also play an important role with regard to adherence to therapy. The final questionnaire consisted of 13 questions (4 concerning demographic information, 9 concerning satisfaction with treatment and adherence to therapy) (Textbox 1) and was validated for language clarity, completeness, and relevance with the help of 10 volunteers having the same characteristics of the final users. Volunteers were asked to answer a series of questions, such as: Was it difficult to understand? Did you understand what this text means? Could this sentence be made better? Were you able to answer the question spontaneously? Is there anything you would like to delete? Is there anything you would like to add? All the volunteers evaluated the questionnaire without difficulty in interpreting the questions and their respective answers, so it was used in the original form during the pilot phase.

**Questionnaire items for adherence to therapy.**

Demographic information

- What is your marital status?
- Whom do you live with?
- What is your highest educational qualification?
- What is or was your source of income?

Satisfaction with treatment and adherence to therapy

- How long have you been on medications for your kidney disease, hypertension, and diabetes (expressed in years)?
- How satisfied are you with your current treatment?
- How well do you think your kidney disease is controlled?
- How often do you experience side effects from medications?
- Does your medication regimen satisfy you in terms of side effects?
- How easy or comfortable did you find your therapy in the past 2 weeks?
- How satisfied are you with the knowledge of your state of health?
- Have you taken the prescribed doses of the medications in the past 2 weeks?
- Why did you not take the prescribed doses?

### Testing of the Integrated Server-App System

The alpha version of the integrated system was tested by internal staff; multiple phases of debugging and implementation of new features were performed to obtain a final product with the planned features and ease of use. The resulting beta version was field tested in Reggio Calabria, Italy, from September 2019 to April 2020 with the help of local GPs. An invitation with the synopsis of the study protocol was sent to 10 GPs, randomly distributed in the urban area of Reggio Calabria, and 4 accepted the invitation and participated in the testing under the supervision of VAP. In order to participate in the testing phase, GPs were asked to randomly recruit, from the entire list of their patients, a minimum of 5 volunteers with the same characteristics of the final users of the system, thus fulfilling the following inclusion criteria: older than 18 years, creatinine 1.5 to 4.0 mg/dL (men) or 1.3 to 3.5 mg/dL (women), taking antihypertensive medications, own a smartphone with Android or iOS operating system (or assisted by family or caregivers), and written informed consent. For patients assisted by family or caregivers, the management of the app, including data entering and receiving of the medication alert, was the responsibility of the caregiver. Patients in other clinical trials or visually impaired or with acute kidney disease, rapidly progressive nephropathies or malignancies, or impaired cognitive abilities were excluded.

To test the platform in a real-life setting, GPs were asked to see the volunteers at the beginning of the testing phase, after 3 months, and after 6 months. During the first visit, patients were invited to download the Smit-CKD app from Google Play or the App Store. At all 3 visits, GPs recorded in the platform clinical data, laboratory data, and current medications (with date and time of administration) and set the frequency of measurement of blood pressure, body weight, and blood glucose (the latter for diabetics only). At each visit, patients were asked to complete the questionnaire about adherence to therapy. Throughout the testing phase, patients received alerts on their app according to GP settings reminding them to measure clinical data and take medications. Registered measurements and feedback on medications taken were sent in real time to the Smit-CKD server via internet connection. Compliance of the volunteers in the use of the app was carefully monitored by their GPs, who checked the amount of clinical data transmitted to the platform and feedback to the received alerts on a weekly basis. In case of missing feedback, the participant was contacted to rule out app or mobile phone malfunction.

### Study Outcomes

The outcomes of this study were user satisfaction, willingness to use the app, and potential usefulness of the integrated system.

Patient satisfaction was determined after the testing phase using a 7-question questionnaire (Textbox 2). We considered the tools available in the literature too complex and time consuming for our participants, so we designed a simpler version for this study. A similar questionnaire was administered to GPs for the web component (Textbox 2). In both questionnaires, satisfaction was expressed on a 5-point scale (1=poor satisfaction and 5= high satisfaction). Willingness to use the app was indirectly measured based on feedback received via the platform (entered measurements, answers to therapy alerts). Potential usefulness of the app was measured in terms of adherence to the therapy. For this purpose, the questionnaire for the adherence to therapy was administered 3 times, at baseline and after 3 and 6 months, and results from each visit were compared.

**Questionnaires administered to patients and general practitioners to measure the satisfaction level in the use of the integrated server-app system.**

Patient satisfaction questionnaire

- How easy is it to use the app?
- How easy do you find connecting the supplied blood pressure monitor to the app?
- How easy do you find it to enter data in the app?
- How useful do you find the alerts that remind you to take the measurements?
- How useful do you find the alerts that remind you to take the medications?
- How often does the app crash or have problems?
- What is your overall opinion of the app?

General practitioner satisfaction

- How easy is it to use the web platform?
- How easy do you find entering data on the web platform?
- How easy do you find searching for information you need from the platform (eg, data entered for a specific patient)?
- How easy do you find the navigation between the various tabs on the platform?
- Was the support provided for use of the platform sufficient?
- How often does the web platform crash or have problems?
- What is your overall opinion regarding the web platform?

### Ethics Approval

The study protocol was approved by the ethical committee of Commissione per l’Etica e l’Integrità nella Ricerca, National Research Council. All participants gave their informed consent. The integrated system was designed in accordance with the current European legislation regarding data processing (EU Regulation 679/2016). Data encryption was applied to protect data in case of accidental release of information; pseudonymization guarantees that personal data cannot be attributed to an identified or identifiable natural person. In addition, to protect data from lawful access to the platform, differentiated log-in credentials were created. To avoid illegal access to the computer system, the adoption of 2-factor authentication was implemented. Finally, the information technology system server location meets the necessary requirements in terms of security, redundancy, and operational recovery in the event of a disaster (disaster recovery).

### Statistical Analysis

Data were expressed as mean and standard deviation for normally distributed variables, median and IQR for nonnormally distributed variables, and frequency and percentage for categorical variables. Clinical data were anonymously extracted from the web platform. Data on adherence to therapy were obtained by entering in the final data set the score of the respective questionnaires. Differences in adherences to therapy across visits (expressed as proportion of adherent patients) were analyzed using the N-1 chi-square test as recommended by Campbell [39] and Richardson [40]. Statistical analysis was performed with open source MedCalc (version 18, MedCalc Software Ltd).

## Results

### Architectural Features and Functionality of the Web Platform

**Figure 1:**
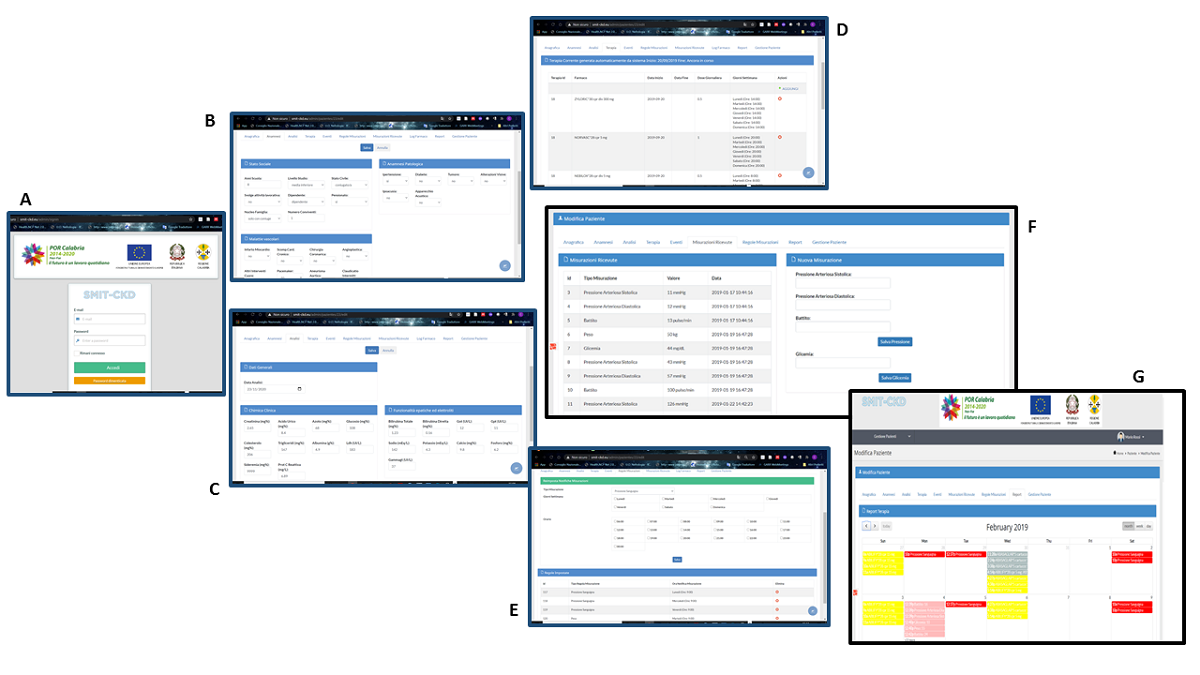
Smit-CKD server: (A) home page/log-in, (B) anamnesis section, (C) laboratory examinations section, (D) therapeutic prescriptions, (E) measurements rules, (F) measurements received by app, (G) feedback received by app: shift from red to pale red indicates patient recorded blood pressure, glucose, or body weight; yellow indicates they took the prescribed medications.

The Smit-CKD server (Figure 1) is accessible via 2-step authentication [41]. Access to the system is granted with 3 profiles (system administrator, medical supervisor, and doctor) with access to all or some tabs of the portal, according to their role. Furthermore, sensitive data can be accessed by the referring GP only.

The server component consists of a series of tabs, each designed to collect medical information. The first 3 tabs are dedicated to patient anamnesis, laboratory measurements, and ongoing therapies. Each medication is selected from a list of pre-entered medications; once the drug is selected, dosage and time of administration can be chosen.

Another tab allows the GP to enter the schedule of measurement to be performed by the patient (blood pressure, weight, blood glucose). Both settings are transmitted to the Smit-CKD app installed on the patient’s smartphone, which sets the appropriate alarms and reminders. Finally, 2 tabs allow GPs to monitor all measurements performed by the patients and transmitted via app and feedback from patients about the medication alarm.

### Architectural Features and Functionality of the Mobile App

**Figure 2:**
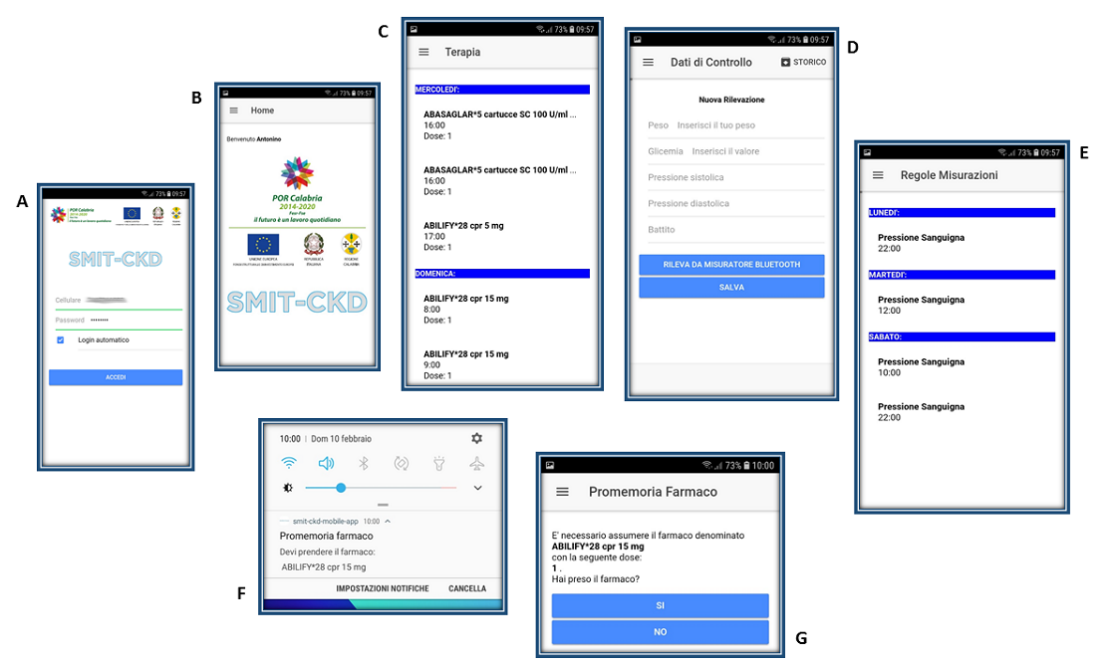
Smit-CKD app: (A) log-in, (B) home page, (C) overview of therapy prescribed by doctor, (D) section for entering data, with overview of old measurements (history section), (E) overview of measurement rules, (F) example of reminder for therapy, (G) patient selects yes or no if they take or do not take the medicine; information is transmitted to doctor via Smit-CKD server, as seen in Figure 1.

The Smit-CKD app (Figure 2) has been developed for Android and iOS. An internet connection allows app and server to communicate. Access is granted by username and password after accepting use conditions. From the menu on the home screen of the Smit-CKD app, users can perform the following tasks:

- Pair the smartphone with a blood pressure monitor equipped with Bluetooth interface (configure blood pressure monitor)
- Display medications prescribed by the doctor (therapy)
- Enter control data, such as weight, blood glucose, and blood pressure, in case no Bluetooth monitor has been paired (control data). Data entered via app are transmitted in real time to the referring GP
- Visualize the history of all measurements taken or entered and consult the report of measurements sent to the remote server (history)

In addition to these activities, the app is designed to send personalized timed notifications to users reminding them to take a prescribed drug or measure weight, blood glucose, or blood pressure. After receiving the notification, the user is required to select YES or NO from the input box to indicate if the required action (for example “take the pill xxx”) has been performed. This allows GPs to monitor the progress of the treatment.

### Test Phase

The integrated system Smit-CKD server and mobile app was tested with the help of 22 participants enrolled by 4 GPs. The main clinical and demographic characteristics are reported in Table 1. Mean age was 70 (SD 11) years, with a male proportion of 59% (13/22). Participants were treated with antihypertensive drugs for a median time of 10 (IQR 6.50-21) years. According to the questionnaires administered during the study, a large majority (19/22, 86%) of the participants were satisfied with the treatment and considered their disease well controlled. Only 9% (2/22) of the participants manifested side effects more than once per month, maintaining a high level of satisfaction. Overall, patients considered the management of medications easy and were satisfied with their knowledge of their disease. Before using the app, 64% (14/22) regularly took all drugs, with forgetfulness the most common cause of missed doses. The rate of regular intake rose to 82% (18/22) after 6 months of using the app (Table 2). However, the difference was not significant (*P*=.18), probably due to the small sample.

The level of compliance with the use of the app was satisfactory, with 16 participants entering clinical data (blood pressure, body weight, and glucose) regularly and sending feedback of drug intake. Among the 6 low compliance users, 2 discontinued app use without giving a reason and 4 were minimally compliant due to technical problems related to an obsolete version of Android installed on their smartphone, which caused frequent malfunctioning of the app.

**Figure 3:**
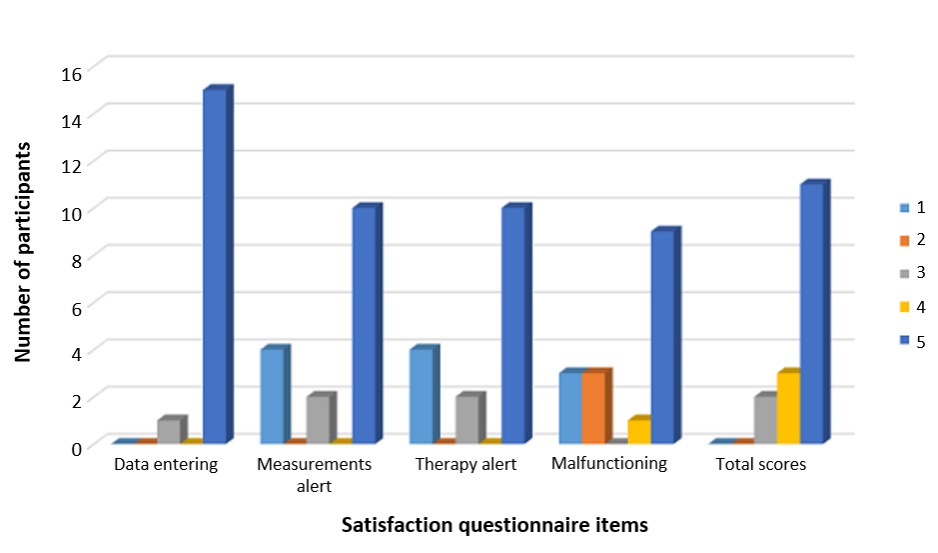
Level of satisfaction of the APP users, expressed on a scale with possible values from 1 to 5 (with 1 equivalent to poor satisfaction - 5 to high approval). Ease of use, data entering, alerts and bugs were considered.

Among users, the percentage of satisfaction was high overall, as shown in Figure 3. The questionnaire administered to GPs to investigate the level of satisfaction with the use of the Smit-CKD server returned even better results, as all the GPs gave a score of 5 to all items in the questionnaire.

**Table 1:** Main demographic and clinical characteristics in the study population (n=22).

			Value
Age (years), mean (SD)			70 (11)
Male, n (%)			13 (59)
Diabetes (yes), n (%)			10 (46)
Cardiovascular comorbidities, n (%)			7 (32)
Cholesterol (mg/dL), mean (SD)			165 (42)
Hemoglobin (g/dL), mean (SD)			13.1 (2.0)
Albumin (g/dL), mean (SD)			4.2 (1.0)
Calcium (mg/dL), mean (SD)			9.4 (0.7)
Phosphate (mg/dL), mean (SD)			3.9 (1.3)
**Creatinine (mg/dL), median (IQR)**			
	Male	2.0 (1.9-2.3)	
	Female	1.9 (1.7-3.2)	
**Marital status, n (%)**			
	Married	14 (64)	
	Widow	6 (27)	
	Separated/divorced	1 (4.5)	
	Never married	1 (4.5)	
**Education level, n (%)**			
	Low/medium education	13 (59)	
	High school diploma	5 (23)	
	University degree	2 (9)	
	No education	2 (9)	
Currently working or retired, n (%)			18 (82)

**Table 2:** Questionnaire responses on adherence to therapy at baseline and after 6 months.

Question			Baseline, n (%)		After 6 months, n (%)
**Satisfaction with the treatment**					
	Satisfied	19 (86)		19 (86)	
	Neutral	2 (9)		2 (9)	
	Unsatisfied	1 (5)		1 (5)	
**Control of kidney disease**					
	Well controlled	17 (77)		17 (77)	
	Neutral	2 (9)		2 (9)	
	Not controlled	3 (14)		3 (14)	
**Side effects**					
	≤1 per month	20 (91)		20 (91)	
	>1 per month	2 (9)		2 (9)	
**Satisfaction with side effects**					
	Highly satisfied	21 (95)		21 (95)	
	Not satisfied	2 (5)		1 (5)	
**Ease of medication regimen**					
	Very difficult	2 (9)		3 (14)	
	Neutral	2 (9)		2 (9)	
	Very easy	18 (82)		17 (77)	
**Knowledge about the disease**					
	Satisfied	17 (77)		17 (77)	
	Neutral	2 (9)		2 (9)	
	Unsatisfied	3 (14)		3 (14)	
**Medication intake**					
	All medication	14 (64)		18 (82)	
	Almost all	4 (18)		2 (9)	
	Part of	4 (18)		2 (9)	
**Reason for missed doses**					
	Forgetfulness	7^a^		3^a^	
	Too many pills	1^a^		1^a^	

^a^Numbers refer only to those who took almost all or some of the medications (baseline n=8 and after 6 months n=4).

## Discussion

### Principal Findings

In this paper we present a new integrated system designed for both GPs and patients consisting of a web-based platform (Smit-CKD server) and an app (Smit-CKD app) with the aim of improving medication compliance and educating patients in self-monitoring of the most common risk factors for CKD and cardiovascular disease. The beta version of the integrated system was tested on a small number of GPs and patients, well representative of the target population, confirming willingness and ease of use; in addition, even with a small sample, our data suggest the potential usefulness for improving adherence to therapy.

Growing coverage of mobile cellular networks has recently made mHealth capable of changing the approach to health systems worldwide. In this scenario, mobile apps are now regarded as potentially useful for changing health behavior and promoting self-management [42,43]. Among apps specifically designed for CKD patients, the *My Kidneys, My Health handbook* provides the user with educational information about detection of kidney disease and advice for a healthier life, including a calculator to compute the individual risk of kidney disease. *H2O Overload: Fluid Control for Heart-Kidney Health* is designed to help fluid intake control. *CKD Go!* allows creation of personalized action plans based on the estimated glomerular filtration rate and urine albumin-creatinine ratio, well known risk factors of CKD progression. CKD patients are often subject to dietary restrictions in order to control the progression of the disease. *My Food Coach*, designed by the National Kidney Foundation, offers nutritional advice from health care professionals. The American Association of Kidney Patients *myHealth Nutrition Guide* is an interactive app that provides the user with the nutrient values of more than 300 commonly consumed foods and many fast food restaurant options. Similarly, *Kidney APPetite* helps monitor daily nutrients and fluid intake. *Wholesome* collects healthy recipes from the web and contains personal recommendations to optimize nutrition. Focused on more specific dietary restrictions, *Oxalator* helps the user following a low oxalate diet, while *Phosphorus Foods Diet Guide* and *MyKidneyDiet – Phosphate Tracker* allows the user to monitor the content of phosphate in their diet.

Markossian et al [44] recently proposed a mobile app for self-management in stage 3-4 CKD patients. Features include the monitoring of clinical parameters, such as weight, blood pressure, and glucose, some aspects related to COVID-19 infection, and medication tracking. In addition, the automated system recognizes values out of range and suggests the patient contact the referring clinician; virtual visits can also be implemented.

The Smit-CKD app adds the self-monitoring of clinical parameters (blood pressure, blood glucose, and body weight), reminders to improve adherence to therapy, and transmission in real time of clinical information to the referring GP, thus allowing continuous monitoring of the health status of the patient and adherence to the medication regimen. This exchange allows the GPs to promptly intervene if data are out of the normal range or adherence to therapy is low; consequently, the patient is responsible for the self-monitoring of the main risk factors of CKD while feeling constantly monitored by their doctor.

### Strengths and Limitations

A strength of the proposed integrated system is good tolerability reported by GPs and users, who appreciated the ease of use. Furthermore, the alerts were helpful in reminding users to take their medications; this was supported by an increase in the adherence to therapy of 18% after 6 months of use.

However, our study has some important limitations. First of all, due to the low number of users and the study design, we cannot prove that this improvement in adherence to therapy is due to the use of the app. Second, even though blood pressure measurements and laboratory exams, useful for CKD monitoring, were collected during the testing phase, the limited number of users and short duration of follow-up prevent us from assessing the real impact of our system on CKD progression and clinical outcomes. However, this paper is not meant for describing the results of a clinical study but rather to introduce the new integrated system and its features developed for patients affected by CKD and their GPs. Finally, user satisfaction was not measured using validated tools but with a new, simpler questionnaire specifically designed for this study.

### Conclusion

Our results suggest that the Smit-CKD app, easy to use and well accepted by patients, may improve adherence to therapy and empower patients affected by CKD. The use of the counterpart Smit-CKD server by GPs showed to be helpful in tracking the evolution of the disease in real time, preventing negative clinical outcomes, and increasing involvement of patients in the management of their clinical condition. The use of the integrated system at a national level could allow a more effective monitoring of patients in the early stages of CKD, contributing to slowing disease progression and delaying the first visit to the nephrology unit. Additional studies designed to test this app in a randomized controlled setting are required to clarify if the use of this integrated system will have long-term effects on medication adherence and if self-monitoring of risk factors will improve clinical outcomes in this population.

## Abbreviations

CKD: chronic kidney disease
GP: general practitioner
mHealth: mobile health
